# Improvements in Insulin Resistance and Glucose Metabolism Related to Breastfeeding Are Not Mediated by Subclinical Inflammation

**DOI:** 10.3390/metabo14110608

**Published:** 2024-11-09

**Authors:** Julia Martins de Oliveira, Patrícia Médici Dualib, Alexandre Archanjo Ferraro, Rosiane Mattar, Sérgio Atala Dib, Bianca de Almeida-Pititto

**Affiliations:** 1 Post-Graduation Program in Endocrinology and Metabology, Federal University of Sao Paulo, Sao Paulo 04022-001, SP, Brazil; juliamoliveira@gmail.com (J.M.d.O.); patricia.dualib@uol.com.br (P.M.D.); sergio.dib@unifesp.br (S.A.D.); 2 Department of Medicine, Federal University of Sao Paulo, Sao Paulo 04022-001, SP, Brazil; 3 Department of Pediatrics, University of São Paulo, Sao Paulo 05403-000, SP, Brazil; ferraro@usp.br; 4 Department of Obstetrics, Federal University of Sao Paulo, Sao Paulo 04023-062, SP, Brazil; rosiane.mattar@unifesp.br; 5 Department of Preventive Medicine, Federal University of Sao Paulo, Sao Paulo 04024-002, SP, Brazil

**Keywords:** gestational diabetes, subclinical inflammation, insulin resistance, breastfeeding

## Abstract

**Background:** Lactation is known to improve insulin resistance, but this phenomenon remains poorly understood. Our goal was to evaluate whether subclinical inflammation could mediate the association between breastfeeding (BF) and improvement in glucose metabolism and markers of insulin resistance (MIRs) in the postpartum. **Methods:** A total of 95 adult women (≥18 years) with a BMI ≥ 25 kg/m^2^ from the outpatient clinic of the Federal University of São Paulo were followed from early pregnancy until 60 to 180 days postpartum. The patients were divided based on their BF status: BF and non-BF groups. A latent variable termed SubInf was created incorporating inflammation-related biomarkers: adiponectin, E-selectin, branched-chain amino acids, zonulin, copeptin, and lipopolysaccharides. The association of BR with MIRs in the postpartum was evaluated through linear regression analysis, and mediation analysis was performed to evaluate the role of SubInf in this association. **Results:** The groups were similar regarding gestational diabetes mellitus (GDM) prevalence, pre-gestational BMI, caloric intake, physical activity, and postpartum weight loss. The BF group presented lower levels of triglycerides (TGs), fasting glucose, fasting insulin, TG/HDLcholesterol ratio (TG/HDL), TyG index, and HOMA-IR compared to the non-BF group. A linear regression analysis adjusted for scholarity, parity, pre-gestational BMI, GDM, weight gain during pregnancy, and mode of delivery revealed an inverse association between BF and fasting glucose [−6.30 (−10.71 to −1.89), *p* = 0.005), HOMA-IR [−0.28 (−0.50 to −0.05), *p* = 0.017], TyG index [−0.04 (−0.06 to −0.01), *p* = 0.002], and TG/HDL ratio [−0.23 (−0.46 to −0.01), *p* = 0.001]. In the mediation analysis, SubInf did not mediate the indirect effect of BF on MIRs. **Conclusions:** In overweight and obese women, an association between BF and improvement in MIRs in the postpartum was seen, corroborating that BF should be stimulated, especially in these cardiometabolic high-risk women. Subclinical inflammation did not seem to mediate this association.

## 1. Introduction

Gestational diabetes mellitus (GDM) is a condition of increasing prevalence worldwide. Brazilian studies have estimated that up to 18% of pregnancies are complicated with GDM [1]. This is worrisome since GDM is a recognized risk factor for the development of type 2 diabetes mellitus (T2DM) later in life, with an estimated nearly tenfold increased risk when compared to non-GDM pregnancies [2,3,4,5]. A meta-analysis of 39 studies evaluated which risk factors were more associated with progression to T2DM and found that a higher pre-gestational body mass index (BMI), a family history of T2DM, non-white ethnicity, an early diagnosis of GDM, increased HbA1c, use of insulin during pregnancy, hypertensive disorders of pregnancy, multiparity, and preterm delivery were the most associated factors [6].

In women with higher chances of developing T2DM, including those with a history of GDM, it is essential to implement postpartum strategies that might help lower the risk. Lactation has been associated with lower glucose levels, improved glucose tolerance and a favorable metabolic profile in the short term, and decreased future risk of metabolic syndrome (MetS) and T2DM in the long term [7,8,9,10,11,12]. The CARDIA study, conducted by Gunderson EP et al., followed women for 20 years after delivery and observed an inverse relation between lactation and insulin resistance syndrome, with a more significant risk reduction among patients with a history of GDM [13]. This same cohort was then followed for an additional 10 years, and after this period of 30 years, the duration of lactation was strongly and inversely related to the incidence of T2DM, irrespective of the diagnosis of GDM [14].

Gunderson EP and colleagues also performed the SWIFT study, which followed 1010 GDM women for nearly 2 years postpartum. They found that both the duration and intensity of lactation were inversely associated with the incidence of T2DM, even after adjustments for age and various maternal and perinatal risk factors [15]. A recent meta-analysis of six studies that included more than 200,000 women suggested that breastfeeding (BF) for more than 12 months is associated with a 30% relative risk reduction of developing T2DM (95% CI 0.78–0.97, *p* = 0.01) [16]. These are some of the studies that suggest that lactation is associated with improvement in metabolic parameters and reduction in future risk of MetS and T2DM. The mechanisms that could explain these effects of breastfeeding are still under investigation [17].

Our group has recently evaluated the role of prolactin in improving markers of insulin resistance (MIRs) in lactating women and did not find a significant mediation effect in this association [18]. Pathways involving other hormone production and reduction in inflammation are possible explanations for the improvements in MIRs observed in the BF population. Studies have described that chronic inflammation leads to the production of cytokines such as TNF-α and IL-6 that activate intracellular pathways, which lead to insulin resistance [19]. Some biomarkers that have been related to inflammation and endothelial dysfunction in previous studies include adiponectin, branched-chain amino acids (BCAAs), E-selectin, copeptin, zonulin, and lipopolysaccharides (LPSs) [20,21,22,23,24,25,26,27,28,29,30,31,32,33,34,35]. Thus, the goal of the present study is to analyze the association of BF with MIRs and the potential mediation effect of these biomarkers in overweight/obesity women with or without GDM.

## 2. Materials and Methods

**Figure 1:**
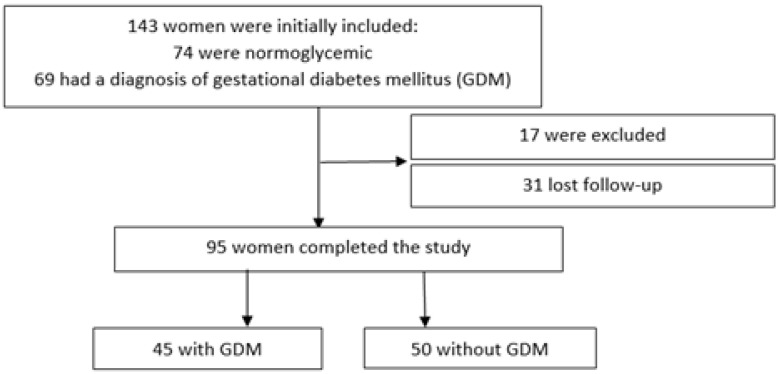
Flowchart graph of our study.

The methodology of this study has been previously described by our group [18]. Briefly, this cohort study included overweight or obese pregnant women from the outpatient Clinic of Obstetrics or the Gestational Diabetes Outpatient Clinic of the Diabetes Center of the Federal University of São Paulo, SP, Brazil. Our sample was obtained through convenience, since all patients that attended these outpatient clinics between September 2018 and December 2019 were invited to participate. Exclusion criteria included pregnancies that were not singleton, women with known chronic diseases, and use of medications (except for those regularly prescribed during pregnancy). A total of 143 women were initially included, 74 of which were normoglycemic and 69 of which had a diagnosis of GDM. Patients were followed throughout pregnancy and until postpartum (60 to 180 days after delivery). In total, 95 women, 45 with GDM, completed our study (Figure 1). Patients with GDM who used insulin during pregnancy had the treatment suspended after delivery. The Federal University of São Paulo ethics committee approved this study (Protocol Number: CAAE: 89108618.0.0000.5505). All participants provided informed consent.

The IAPDSG criteria was used to diagnose GDM [36,37]. Only women with first-trimester fasting plasma glucose greater than 100 mg/dL or at least two altered points in the 75 g oral glucose tolerance test (OGTT) (≥92, ≥180, ≥153 mg/dL at 0, 60, and 120 min, respectively) were included. These stricter criteria were applied to exclude borderline cases of GDM so that a better comparison could be made between insulin-resistant and normoglycemic controls.

Participants were followed up during pregnancy and postpartum (at 60 to 180 days after delivery). Trained interviewers applied structured, standardized questionnaires throughout pregnancy and at postpartum to obtain socio-demographic and medical data.

All participants underwent evaluations that included anthropometric measures (weight and body mass index) and blood pressure assessment. Patients recorded all foods and beverages consumed over three days in a standardized form. Registration was made on alternate days and included at least one weekend day [38]. The Diet Pro software was used to calculate the total energy value of macro- and micronutrients, based on the Brazilian Food Composition Table (TBCA) as a reference [39].

After an overnight fast, an oral glucose tolerance test (OGTT) was performed, and blood was drawn at 0 and 120 min. The samples were immediately centrifuged and analyzed by a private certificate laboratory. Plasma glucose was determined by the glucose oxidase method. Total cholesterol, HDL-c, and triglyceride concentrations were measured by enzymatic colorimetric methods and processed in an automatic analyzer. LDL-c and VLDL-c concentrations were obtained by difference using the Friedewald equation. E-selectin (Human SELE Elabscience-E-EL-H0876; Houston, TX, USA), zonulin (Human Zonulin Elabscience-E-EL-H5560; Houston, TX, USA), adiponectin (Human ADP/Acrp30 Elabscience-E-EL-H6122; Houston, TX, USA), copeptin (Human CPP Elabscience-E-EL-H0851; Houston, TX, USA) and lipopolysaccharides (LPS—Human LBP Elabscience-E-EL-H6108; Houston, TX, USA) were measured using enzyme-linked immunosorbent assay (ELISA Kit, Elabscience Kit; Houston, TX, USA), while branched-chain amino acids (BCAAs) were assessed using High-Performance Liquid Chromatography (MBS169297, HPLC, My Biosource; San Diego, CA, USA).

Insulin resistance was assessed using HOMA-IR (homeostasis model assessment—insulin resistance) [40], the triglycerides–glucose index (TyG Index) [41,42], the triglyceride-to-HDL-cholesterol ratio (TG/HDL), and the metabolic score for insulin resistance (Met-IR) [43]. The following equations were used:

$$\mathrm{HOMA}-\mathrm{IR}=\frac{\left[\mathrm{fasting}\;\mathrm{Insulin}\left(\mu \frac{\mathrm{mU}}{L}\right)\times \mathrm{fasting}\;\mathrm{Glucose}\left(\frac{\mathrm{mmol}}{L}\right)\right]}{22.5}$$

$$\mathrm{TyG}\;\mathrm{index}\;=\frac{\ln[(\;\mathrm{fasting}\;\mathrm{triglycerides}\;(\mathrm{mg}/\mathrm{dl})\times \;\mathrm{fasting}\;\mathrm{Glucose}\;\left(\mathrm{mg}/\mathrm{dl}\right)]}{2}$$

$$\mathrm{Met}-\mathrm{IR}\;=\frac{\ln[2\times \;\mathrm{glucose}\;(+\;\mathrm{triglycerides}\;\left(\frac{\mathrm{mg}}{\mathrm{dL}}\right)]\times \;\mathrm{BMI}\;\left(\frac{\mathrm{kg}}{m^{2}}\right)}{\ln[\;\mathrm{HDLc}\;\left(\frac{\mathrm{mg}}{\mathrm{dL}}\right)]}$$

HOMA-β (homeostasis model assessment of β function) was calculated using the following equation [40]:

$$\mathrm{HOMA}-\;\beta =\frac{\left[20\times \;\mathrm{fasting}\;\mathrm{insulin}\;\left(\frac{\mu \mathrm{UI}}{\;\mathrm{mL}}\right)\right]}{\left[\;\mathrm{fasting}\;\mathrm{glucose}\;\left(\frac{\mathrm{mmol}}{\;L}\right)-3.5\right]}$$

### 2.1. Variable Definitions

Breastfeeding (BF) status, the exposure variable, was assessed during the postpartum visit. The mother was inquired about the duration of BF, whether she was still breastfeeding during the postpartum visit, and if any other liquids or foods (such as water, tea, or fruits) had been introduced. BF was classified as predominant or exclusive if breastmilk remained the baby’s primary source of nutrition even if water, tea, or fruits were also given (excluding formula or cow’s milk).

The outcome variables included those related to glucose metabolism and insulin resistance, such as fasting glucose and insulin, as well as markers of insulin resistance (MIRs). These markers included HOMA-IR, the TG/HDL cholesterol ratio, the TG and glucose (TyG) index, and the Met-IR.

A latent variable termed “subclinical inflammation” (SubInf) was created. This variable cannot be directly measured and is inferred indirectly through a mathematical model from the observable subclinical inflammation variables: E-selectin, branched-chain amino acids (BCAAs), lipopolysaccharides (LPSs), zonulin, adiponectin, and copeptin. By aggregating these parameters into one variable, the underlying concept of subclinical inflammation can be more robustly evaluated. The theoretical model including this latent variable is represented in the supplemental material (S2).

### 2.2. Statistical Analysis

To compare clinical and laboratory variables, as well as maternal–fetal outcomes based on BF status, the Student *t*-test or Mann–Whitney test (for continuous variables) and the Chi-squared test (for categorical variables) were employed. The associations between BF (exposure) and outcomes related to glucose metabolism and insulin resistance—grouped as MIR, which includes HOMA-IR, TG/HDL-c, TyG index, and Met-IR—were analyzed through linear regression with adjustments for potential confounders identified with the use of Directed Acyclic Graphs (DAGs). A DAG is a visual representation derived from a theoretical mathematical model that illustrates the relationships between variables. Its purpose is to pinpoint the essential adjustment variables needed to prevent biases or overadjustments [26,27]. DAG was used in the mediation analysis to evaluate potential confounders in the relationship between BF and glucose tolerance. In our model, the minimal sufficient adjustment set for estimating the total effect of BF on glucose tolerance comprised education level, parity, type of delivery, GDM, pre-gestational BMI, and weight gain during pregnancy. The DAGitty software, version 3.0 (www.dagitty.net), accessed on 9 October 2023, was used to create the DAG model [44]. The final DAG model that associates BF with glucose metabolism and MIRs is shown in the Supplemental Material (Figure S1). The mediation analysis was also adjusted for these variables.

Mediation analyses were conducted to evaluate the total, direct, and indirect effect of BF on MIRs, with subclinical inflammation considered as a mediator. A latent variable named SubInfwas constructed to represent subclinical inflammation. A latent variable is not directly observable but is inferred from a set of related observable indicators [45]. It is obtained through the regression function of observed variables. The selected indicators in our study included E-selectin, BCAAs, LPSs, zonulin, adiponectin, and copeptin, which were chosen due to their ability to capture different aspects of the latent variable, such as inflammation and endothelial dysfunction. To construct the latent variable, a structural equation modeling approach was employed. This method allowed for the extraction of the underlying structure of variability in the observed indicators and the estimation of weights or coefficients contributing to the formation of the latent variable (Supplementary Figures S2 and S3). The latent variable significantly contributes to a deeper understanding of the phenomenon in question (subclinical inflammation) in a better way than using each biomarker separately.

For statistical analysis, the Statistical Package for the Social Sciences^®^, for Windows version 22.0 (2013, IBM Corp., Armonk, NY, USA), and the Statistics and Data Science (STATA^®^), version 17.0 (StataCorp. 2021. Stata Statistical Software: Release 17. College Station, TX, USA: StataCorp LLC.), were used. The mediation analysis was performed using the command medeff, and the latent variable SubInf was created using the statistical software STATA^®^. *p* was considered statistically significant if <0.05.

## 3. Results

Two groups were defined according to the participant’s BF status at the postpartum visit: BF (*n* = 44) and non-BF (*n* = 51), totaling 95 participants. Among the 44 participants in the BF group, 11 were providing their babies additional nutrients and liquids (such as water, tea, and fruits), but breast milk remained the primary source of calories, and no formulas or cow’s milk were given to babies in this group. The BF and the non-BF groups had similar socio-demographic characteristics, familial history, current life habits, and pre-gestational BMI (Table 1), as reported in a previous publication [18].

In the postpartum assessment, the BMI was comparable between the groups; however, the non-BF group experienced greater weight gain during pregnancy. The proportion of physically active women and the total daily caloric intake were similar across both groups.

The markers of insulin resistance (TG/HDL, TyG index, and HOMA-IR) were lower in the BF group compared to the non-BF group (Table 2), whereas Met-IR was similar among groups.

Subclinical inflammation parameters were compared separately between groups, and only LPS levels were different, with lower levels being found in the BF group [6.8 (4.2–10.6) vs. 9.2 (6.9–11.5), *p* = 0.048] when compared to the non-BF group. Adiponectin, BCAA, E-selectin, copeptin, and zonulin were not statistically different between groups (Table 1).

To evaluate the association between BF and MIR/glucose levels, a linear regression analysis was performed and published elsewhere [17]. As showed previously, in the unadjusted model, fasting blood glucose, fasting insulin, HOMA-IR, TyG index, and TG/HDL ratio were inversely associated with BF. With the inclusion of the parameters indicated by the DAG model—parity, scholarity, pre-gestational BMI, weight gain during pregnancy, GDM, and mode of delivery—an inverse association was found between BF and fasting glucose [−6.30 (−10.71 to −1.89), *p* = 0.005), HOMA-IR [−0.28 (−0.50 to −0.05), *p* = 0.017], TyG index [−0.004 (−0.06 to −0.01), *p* = 0.002], and TG/HDL ratio [−0.23 (−0.46 to −0.01), *p* = 0.001] (Table 2).

A mediation analysis was performed to determine the total and direct effect of BF and the indirect effect of BF, via SubInf, on MIR and glucose levels. BF had a total effect on fasting blood glucose [−6.20 (−10.80 to −1.61)], fasting insulin [−0.22 (−0.44 to −0.0002)], HOMA-IR [−0.28 (−0.52 to −0.05)], TyG index [−0.03 (−0.06 to −0.01)], and TG/HDL ratio [−0.24 (−0.48 to −0.08)]. SubInf did not mediate the effects in either parameter (Table 3).

## 4. Discussion

In high-cardiometabolic-risk women (overweight/obese and/or those with a past medical history of GDM), BF for as little as two to six months was linked to improved glucose metabolism and insulin resistance profiles. This was evidenced by measures such as fasting blood glucose, fasting insulin, TG levels, TG/HDL ratio, TyG index, and HOMA-IR, even after adjustments for potential confounders. It is worth noting that the groups were largely homogeneous in most parameters, including potential confounding factors like daily caloric intake, physical activity, and postpartum weight loss. Despite the non-BF group experiencing greater weight gain during pregnancy, weight loss and final weight at the postpartum visit were similar across groups. This homogeneity strengthens the hypothesis that lactation itself played a physiological role in the improvement in MIRs.

These results corroborate other reports from the literature that describe the benefits of BF on glucose metabolism [7,8,15,16,46,47,48]. Mechanisms to explain this phenomenon are still lacking and are most probably complex and multifactorial. Our hypothesis was that BF could be associated with lower subclinical inflammation and that this could be a potential mechanism behind the improvement in glucose metabolism and MIRs.

To test our hypothesis, we first compared the six biomarkers related to subclinical inflammation and endothelial disfunction (E-selectin, BCAAs, LPSs, zonulin, adiponectin, and copeptin) among the BF and non-BF groups. LPS levels were significantly higher in the non-BF group when compared to the BF group, whereas other biomarkers were not statistically different between the groups.

Studies comparing the levels of these biomarkers during pregnancy and the puerperium have mixed results in the literature.

A study conducted in humans during the first and third trimesters of pregnancy compared the gut microbiota in these two periods in healthy women and described that, by the third trimester, there was a loss of within-individual bacterial diversity, irrespective of maternal weight and diet [49]. A decrease in bacterial diversity has been associated with dysbiosis that increases LPS translocation and metabolic diseases such as obesity and T2DM [50]. Interestingly, prolactin was shown to inhibit the secretion of proinflammatory cytokines induced by LPSs in fetal membranes in a study conducted by Flores-Espinosa et al. [51].

In our study, we found significantly higher levels of LPSs and lower levels of prolactin in the non-BF group when compared to the BF group. It is important to say that weight was similar among groups, as was daily caloric intake. We did not, however, compare the content of fat in the diets of the groups. We found no publications comparing the levels of LPSs in puerperium women according to their lactation status, but we speculated that higher prolactin levels in the BF group could have mitigated the deleterious proinflammatory effects of LPSs.

Serum zonulin levels have also been found to be increased in the context of increased intestinal permeability and are associated with insulin resistance and obesity, possibly mediated by an increase in IL-6 production [34]. In our study, however, the groups had similar levels of zonulin. To the best of our knowledge, no studies specifically regarding lactation and zonulin levels have been published.

In accordance with our findings, a study conducted by Stueve AM et al. compared adiponectin levels in women at three years’ postpartum and did not find statistically different levels according to duration of lactation [52,53]. It is important to say that the prevalence of GDM was similar among the groups. Although adiponectin levels and the prevalence of GDM were similar across our groups, the short duration of our follow-up complicates comparisons with the previously described data.

Differing from our results, a study conducted by Much D et al.described that GDM women that had been BF for more than 3 months had BCAA levels at 30 min of the OGTT significantly lower when compared to those that had breastfed for less than 3 months [54]. HOMA-IR was positively associated with BCAA levels in their analysis after adjustment for lactation duration. In our study, BCAA levels were comparable across the groups; however, differences between the two studies make direct comparisons challenging. Our population was not composed only of GDM women, and our BF follow-up was very short (only 2 to 6 months), differently from the study by Much D. et al., which has a median follow-up time of 3.6 years. For the other biomarkers, no studies were found associating their levels to glucose metabolism or MIRs.

A latent variable termed “subclinical inflammation” (SubInf), which is a mathematical model inferred indirectly from the observable biomarkers previously described, was created. The idea is that the SubInf variable is a more robust parameter to evaluate the potential mediation effect of inflammation parameters on MIRs. A statistical analysis, however, did not demonstrate that SubInf mediated the improvement in MIRs related to BF.

The physiological mechanisms underlying the improvement in glucose metabolism and MIRs associated with breastfeeding remain unclear, necessitating further research to clarify these processes. In our previous work, we analyzed the potential mediation effect of prolactin on the same parameters as the ones described herein, but prolactin did not mediate the improvement in glucose metabolism or in MIRs [18]. Recent metabolomic studies may contribute to a better understanding of the process. Some authors have suggested that intensive BF is associated with modifications in the lipid metabolism that may exert beneficial effects on maternal metabolic health, but only while lactation is maintained. These changes include downregulation of triacylglycerols and diacylglycerols and upregulation of phospholipids and sphingolipids, a profile that is consistent with suppression of endogenous lipogenesis [4,55]. Another possible mechanism is related to the increased production of oxytocin in lactating women. Oxytocin is a hormone primarily produced during lactation that promotes lipolysis, enhances the adipogenesis of brown adipose tissue, and decreases the deposition of visceral and liver fat [56,57]. It also increases glucose uptake in peripheral organs [58] and stimulates insulin secretion. These effects may exert beneficial effects on glucose metabolism and may decrease insulin resistance. Finally, insulin resistance may also be associated with fibrinolytic dysfunction, as suggested in a study by Morimitsu LK et al. [59].

We recognize limitations in our study. Our sample size was small, and only one blood sample was analyzed in the postpartum period. The duration of BF was short, only 2 to 6 months. Even though the protective effects of BF on the future risk of T2DM have been described after one to six months of lactation [60], it is not possible to discard that different results could have been found if longer periods of time were analyzed. Also, we did not have a formal criterion to evaluate breastfeeding regarding intensity and duration, but we considered that the small babies were breastfeeding on demand, so there was no possible way to interfere with this parameter. It is also important to notice that our population was composed exclusively of overweight or obese women. These factors could influence subclinical inflammation chronically, even in the pre-gestational period. Possibly, if we included lean women in the analysis, the results could have been different, so BF would not be sufficient to improve an inflammatory profile in this group of obese and insulin-resistant women.

One of this study’s strengths is the homogeneity observed between the groups in several factors that could have influenced the results, such as daily caloric intake, physical activity, and postpartum weight loss, despite the small sample size. This similarity between the groups reinforces our hypothesis that lactation played a role in improving glucose levels and insulin resistance markers (MIRs). The use of Directed Acyclic Graphs (DAGs) is also a point that should be mentioned, since it strengthens our theoretical concept and avoids overadjustments. Another relevant aspect is that biomarkers of inflammation and endothelial dysfunction are difficult to measure and represent a small part of the total phenomenon of inflammation. In this context, the latent variable is a more robust way to evaluate subclinical inflammation than each biomarker separately. However, it is important to notice that other biomarkers can be evaluated in future studies.

To the best of our knowledge, this is the first study to assess the mediating effect of subclinical inflammation on improvements in glucose metabolism and MIRs in overweight/obese lactating women with and without GDM. Further studies are needed to better clarify the potential mechanisms through which BF improves glucose metabolism and insulin resistance.

## 5. Conclusions

In conclusion, we found that exclusive or predominant BF, even when adjusted for several confounders, was associated with improvements in glucose metabolism and markers of insulin resistance in postpartum women with a high risk for glucose intolerance, such as those who are overweight, obese, and/or have a diagnosis of GDM. This finding is in accordance with what has been described by other studies and corroborates the importance of stimulating BF. Subclinical inflammation did not mediate the association herein described.

## Figures and Tables

**Table 1:** Participants’ data throughout pre-gestation, third trimester, and postpartum periods, according to breastfeeding status.

	Breastfeeding		
	No	Yes	*p*
Pre-Gestational Period			
Age (years)	30.3 (7.3)	31.1 (5.8)	0.560
Race, white, *n* (%)	22 (43.1)	22 (50.0)	0.717
High educational level, *n* (%)	12 (23.5)	14 (31.8)	0.366
3 or more previous gestations, *n* (%)	18 (35.3)	17 (38.6)	0.736
Diabetes mellitus among family members, *n* (%)	18 (35.3)	15 (34.1)	0.902
Physical activity before pregnancy, *n* (%)	23 (45.1)	17 (38.6)	0.525
Pre-gestational weight (kg)	76.3 (12.0)	78.3 (11.4)	0.418
Pre-gestational BMI (kg/m^2^)	29.3 (4.0)	30.4 (3.9)	0.176
BMI ≥ 30 kg/m^2^, *n* (%)	17 (33.3)	20 (45.5)	0.227
**3rdtrimester**			
Physical activity, *n* (%)	2 (4.4)	2 (5.3)	0.862
Smoking, *n* (%)	0	0	-
Alcohol consumption, n (%)	0	0	-
Insulin use during pregnancy, *n* (%)	12 (4.0)	14 (32.6)	0.359
Hypertension during pregnancy, *n* (%)	7 (13.7)	6 (13.6)	0.990
GDM, n (%)	20 (39.2)	25 (56.8)	0.087
Pre-eclampsia, *n* (%)	3 (5.9)	1 (2.3)	0.621
**Postpartum period**			
Weeks of gestation at birth	38.9 (1.2)	38.5 (1.2)	0.128
Vaginal birth, *n* (%)	27 (52.9)	26 (59.1)	0.547
Birth weight (kg)	3.4 (0.4)	3.3 (0.5)	0.303
Weight gain during pregnancy (kg)	11.6 (5.5)	8.1 (6.2)	0.006
Physical activity at postpartum, *n* (%)	2 (3.9)	2 (4.5)	0.880
Diet—daily calorie consumption (kcal)	1743.3 (1604.8–2382.1)	1799.8 (1421.5–2501.4)	0.737
BMI (kg/m^2^)	29.8 (4.4)	29.5 (3.6)	0.723
Waist circumference (cm)	93.4 (9.9)	93.5 (7.8)	0.940
Weight variation at postpartum (kg)	−9.3 (5.4)	−9.5 (4.1)	0.811
Fasting blood glucose (mg/dL)	93.9 (12.6)	89.0 (8.0)	0.036
2h post-OGTT blood glucose (mg/dL)	112.8 (37.8)	103.5 (27.3)	0.185
HbA1c (%)	5.5 (0.4)	5.5 (0.3)	0.880
Fasting serum insulin (µU/mL) ^#^	12.5 (7.7–17.0)	10.0 (6.3–11.6)	0.048
HOMA-IR ^#^	2.6 (1.6–3.9)	2.0 (1.3–2.7)	0.025
HOMA-Beta ^#^	125.8 (95.8–174.5)	121.4 (99.6–188.4)	0.675
TG/HDL ratio ^#^	2.4 (1.3–3.3)	1.8(1.1–2.2)	0.038
TyG index ^#^	4.58 (4.40–4.80)	4.43 (4.32–4.64)	0.005
Met-IR	42.92 (37.73–49.59)	41.65 (36.78–46.14)	0.339
Adiponectin (μg/mL) ^#^	10.7 (5.7–17.9)	9.4 (4.1–13.0)	0.252
BCAA (umol/L) ^#^	570.1 (352.7–777.0)	596.0 (356.6–842.8)	0.669
E-selectin (ng/mL) ^#^	55.7 (47.4–63.0)	54.1 (33.2–62.1)	0.184
Copeptin (pmol/L)	1.5 (0.7)	1.5 (0.6)	0.8660
Zonulin (ng/mL) ^#^	41.0 (35.8–43.7)	40.5 (28.8–42.0)	0.062
LPS (ng/mL) ^#^	9.2 (6.9–11.5)	6.8 (4.2–10.6)	0.048

Data are expressed as mean (standard deviation) when distribution is normal or as median (interquartile interval) ^#^ when distribution is non-normal. Categoric variables are presented as n (percentile). ^#^ Non-normal variables were log-transformed for statistical analysis. Student *t*-test was used for continuous variables and Chi-squared test for categorical variables. Definitions: physically active if >150 min of exercise per week was performed; high educational level present if at least 14 years of schooling were completed. Abbreviations: BMI, body mass index; TG, triglyceride; HDL, HDL-cholesterol; HbA1c, glycated hemoglobin; GDM, gestational diabetes mellitus; OGTT, oral glucose tolerance test; Met-IR, metabolic score for insulin resistance; BCAA, branched-chain amino acid; LPS, lipopolysaccharide. Formulas used to calculate markers of insulin resistance: HOMA-IR (homeostatic model assessment for insulin resistance): [fasting insulin (µmUI/L) ×fasting glucose (mmol/L)]/22.5; HOMA-Beta (homeostasis model assessment of β function): fasting insulin (uUI/mL) × 20/(fasting glucose (mg/dL) × 0.0555)−3.5; triglyceride and glucose (Tyg) index: ln[(TG mg/dL × glucose mg/dL)]/2; Met-IR (metabolic score for insulin resistance): [ln(2 × glucose(mg/dL) + TG (mg/dL)) × BMI (kg/m^2^)]/ln(HDL(mg/dL).

**Table 2:** Linear regression analysis considering MIR variables as dependent variables and breastfeeding as the main independent variable of interest.

	β	IC 95%	*p*
Fasting blood glucose	−6.30	−10.71 to −1.89	0.005
Fasting serum insulin	−0.21	−0.42 to 0.00	0.052
Met-IR	−1.36	−3.00 to 0.28	0.102
TyG index	−0.04	−0.06 to −0.01	0.002
TG/HDL ratio	−0.23	−0.46 to −0.01	0.044
HOMA-IR	−0.28	−0.50 to −0.05	0.017

Model adjusted for scholarity, pre-gestational BMI, parity, type of delivery, GDM, and weight gain during pregnancy. Formulas used to calculate markers of insulin resistance: HOMA-IR (homeostatic model assessment for insulin resistance): [fasting insulin (µmUI/L) ×fasting glucose (mmol/L)]/22.5; triglyceride and glucose (Tyg) index: ln[(TG mg/dL × glucose mg/dL)]/2; Met-IR (metabolic score for insulin resistance): [ln(2 × glucose(mg/dL) + TG (mg/dL)) × BMI (kg/m^2^)]/ln(HDL(mg/dL).

**Table 3:** Mediation analysis considering MIR (fasting blood glucose, fasting serum insulin, HOMA-IR, TyG index, and TG/HDL ratio) as dependent variables, breastfeeding as the interest independent variable, and subclinical inflammation as potential mediator.

	Direct Effect		Indirect Effect		Total Effect		% of Total Effect-Mediated	
	β	IC 95%	β	IC95%	β	IC 95%	β	IC 95%
Fasting glucose	−6.56	−11.19 to −2.07	0.36	−0.46to1.58	−6.20	−10.80 to−1.61	−0.06	−0.21 to−0.33
Fasting Insulin ^#^	−0.19	−0.42 to 0.02	−0.02	−0.08to 0.15	−0.22	−0.44 to −0.0002	0.10	0.04 to 0.68
HOMA-IR ^#^	−0.26	−0.50 to −0.28	−0.02	−0.08 to 0.02	−0.28	−0.52 to−0.05	0.07	0.04 to 0.33
TyG index ^#^	−0.03	−0.06 to −0.01	−0.003	−0.009 to 0.001	−0.04	−0.06 to −0.01	0.07	0.04 to 0.18
TG/HDL ratio ^#^	−0.21	−0.45 to 0.17	−0.22	−0.08to 0.20	−0.24	−0.48 to −0.01	0.09	0.04 to 0.59
Met-IR	−1.44	−4.44 to 1.53	−0.01	−0.71 to 0.66	−1.45	−4.34 to 1.52	0.006	−0.10 to 0.09

**^#^** Values of outcomes were log-transformed for analyses. Fasting glucose is expressed in mg/dL.Formulas used to calculate markers of insulin resistance: HOMA-IR (homeostatic model assessment for insulin resistance): [fasting insulin (µmUI/L) × fasting glucose (mmol/L)]/22.5; triglyceride and glucose (Tyg) index: ln[(TG mg/dL × glucose mg/dL)]/2; Met-IR (metabolic score for insulin resistance): [ln(2 × glucose(mg/dL) + TG (mg/dL)) × BMI (kg/m^2^)]/ln(HDL(mg/dL).

## Data Availability

The raw data supporting the conclusions of this article will be made available by the authors upon request.

## Supplementary Materials

The following supporting information can be downloaded at: https://www.mdpi.com/article/10.3390/metabo14110608/s1, Figure S1: Direct Acyclic Graph for glucose metabolism and markers of insulin resistance in postpartum.; Figure S2: Theoretical model for the investigation of the total, direct, and indirect effect (mediated by biomarkers of inflammation and represented by the latent variable subclinical inflammation, SubInf) of BF on glucose metabolism and insulin resistance in the postpartum. Figure S3: Structural Equation Modeling (SEM), with the observed variables that are linked to the latent variable through factor loadings and the measured errors (e).
