# Supplementary material for: Improvements in Insulin Resistance and Glucose Metabolism Related to Breastfeeding Are Not Mediated by Subclinical Inflammation
Source: Metabolites. 2024 Nov 9;14(11):608. doi: 10.3390/metabo14110608 (PMC11596560; doi:10.3390/metabo14110608)
Supplement: Supplementary file 1 [file metabolites-14-00608-s001.zip › metabolites-3233400-supplementary.pdf]

**Supplementary Materials:**

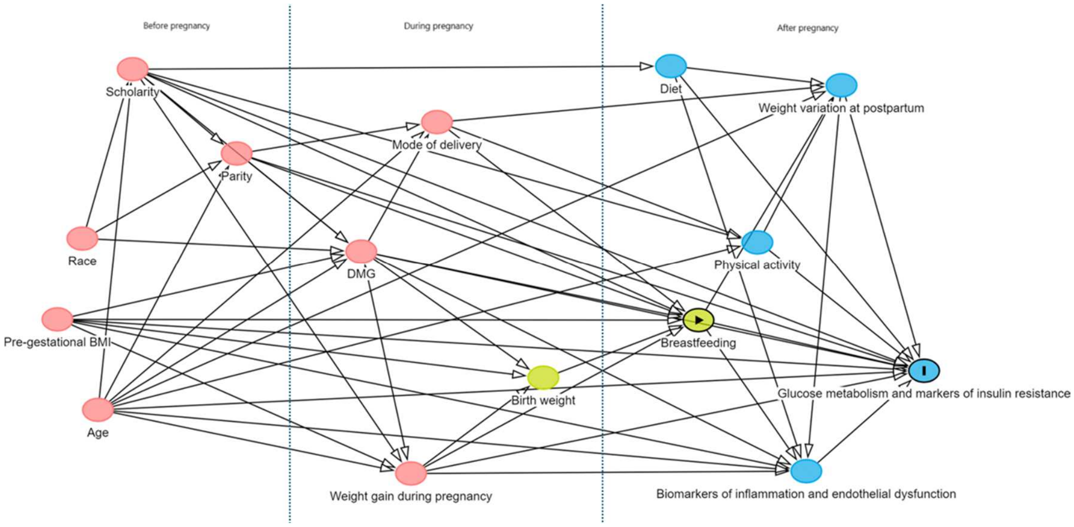


**FigureS1.** Direct Acyclic Graph for glucose metabolism and markers of insulin resistance in postpartum. Arrows represent direct causal effects. "Breastfeeding" is the exposure variable and "glucose metabolism and markers of insulin resistance" is the outcome variable. Variables in pink represent ancestors of exposure and outcome, whereas variables in blue represent ancestor of outcome and variables in green, ancestor of exposure. Created by DAGitty software, version 3.0. Minimal sufficient adjustments for estimating total effect of breastfeeding on glucose metabolism and markers of insulin resistance: gestational diabetes mellitus (GDM), mode of delivery, parity, scholarity, pre-gestational BMI, and weight gain during pregnancy.

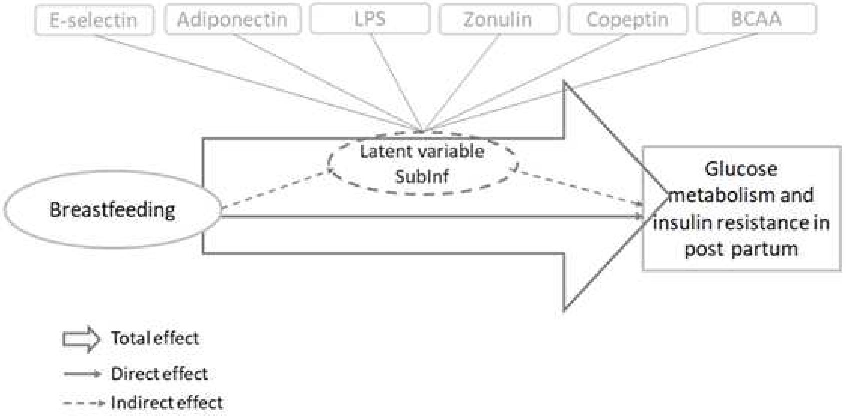


**FigureS2.** Theoretical model for the investigation of the total, direct, and indirect effect (mediated by biomarkers of inflammation and represented by the latent variable subclinical inflammation, SubInf) of BF on glucose metabolism and insulin resistance in the postpartum.

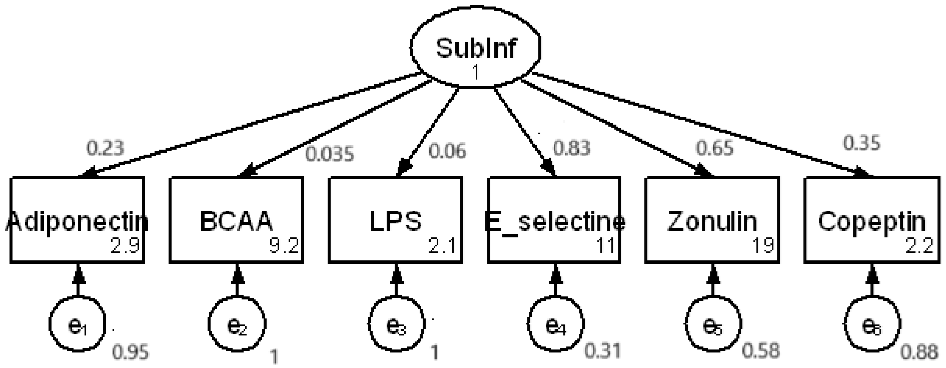


**FigureS3.** Structural Equation Modeling (SEM), with the observed variables that are linked to the latent variable through factor loadings and the measured errors (e).
